# Effect of Ferulic Acid, a Phenolic Inducer of Fungal Laccase, on 26S Proteasome Activities In Vitro

**DOI:** 10.3390/ijms21072463

**Published:** 2020-04-02

**Authors:** Anita Swatek, Magdalena Staszczak

**Affiliations:** Department of Biochemistry and Biotechnology, Institute of Biological Sciences, Maria Curie-Skłodowska University, Akademicka 19, 20-033 Lublin, Poland; anita.swatek@poczta.umcs.lublin.pl

**Keywords:** proteasome inhibitors, phenolic acids, proteolysis, proteolytic enzymes, laccase, lignin-degrading fungi, ligninolytic enzymes, Basidiomycetes

## Abstract

The 26S proteasome is an ATP-dependent protease complex (2.5 MDa) that degrades most cellular proteins in Eukaryotes, typically those modified by a polyubiquitin chain. The proteasome-mediated proteolysis regulates a variety of critical cellular processes such as transcriptional control, cell cycle, oncogenesis, apoptosis, protein quality control, and stress response. Previous studies conducted in our laboratory have shown that 26S proteasomes are involved in the regulation of ligninolytic enzymes (such as laccase) in white-rot fungi in response to nutrient starvation, cadmium exposure, and ER stress. Laccases are useful biocatalysts for a wide range of biotechnological applications. The goal of the current study was to determine the effect of ferulic acid (4-hydroxy-3-methoxycinnamic acid), a phenolic compound known to induce some ligninolytic enzymes, on proteasomes isolated from mycelia of the wood-decomposing basidiomycete *Trametes versicolor*. The peptidase activities of 26S proteasomes were assayed by measuring the hydrolysis of fluorogenic peptide substrates specific for each active site: Suc-LLVY-AMC, Z-GGR-AMC and Z-LLE-AMC for chymotrypsin-like, trypsin-like, and caspase-like site, respectively. Ferulic acid affected all peptidase activities of the 26S fungal proteasomes in a concentration-dependent manner. A possible inhibitory effect of ferulic acid on peptidase activities of the 26S human proteasomes was tested as well. Moreover, the ability of ferulic acid to inhibit (at concentrations known to induce laccase activity in white-rot fungi) the rate of 26S proteasome-catalyzed degradation of a model full-length protein substrate (β-casein) was demonstrated by a fluorescamine assay and by a gel-electrophoretic analysis. Our findings provide new insights into the role of ferulic acid in lignin-degrading fungi. However, the detailed molecular mechanisms involved remain to be elucidated by future studies.

## 1. Introduction

In Eukaryotes, the bulk of intracellular proteins are degraded by a highly selective non-lysosomal pathway that requires the 26S proteasome, a 2.5 MDa ATP-dependent multifunctional protease complex [1,2,3,4]. The 26S proteasome consists of a proteolytic core (called the 20S proteasome) [5,6] capped with one or two 19S regulatory complexes [7], which confer ATP- and ubiquitin-dependence on substrate proteolysis by the proteasome. The barrel-shaped 20S catalytic complex is formed by 28 subunits, arranged into four heteroheptameric rings—with two distal rings of α-type subunits and two inner rings of β-type subunits (α_1–7_β_1–7_β_1–7_α_1–7_). Each β ring contains three distinct proteolytically active centers: chymotrypsin-like (CHTL, β_5_ subunit), trypsin-like (TL, β_2_ subunit), and caspase-like (CL, β_1_ subunit) [8,9]. These active sites preferentially cleave peptide bonds on the carboxyl side of hydrophobic, basic, and acidic residues, respectively. Unlike any other protease, all the proteolytic sites in the proteasomes utilize N-terminal threonines of β-subunits as the active site nucleophiles [10,11]. Substrates enter the 20S proteasome through a narrow channel formed by the α subunits, whose N termini function as a gate [12]. The 26S proteasome, which is present both in the cytosol and nucleus, typically recognizes protein substrates marked for degradation by covalent modification with a polyubiquitin chain [13]. In eukaryotes, ATP is required both for ubiquitin conjugation to substrates and for the functioning of the 26S proteasome, which catalyzes the breakdown of the ubiquitinated and certain nonubiquitinated proteins [14,15]. Ubiquitin conjugation (called ubiquitination or ubiquitilation) is catalyzed by the sequential action of the ubiquitin-activating enzyme (E1), ubiquitin-conjugating enzymes (E2s), and ubiquitin ligases (E3s). The ubiquitin-proteasome pathway (UPP) regulates a variety of critical cellular processes such as transcriptional control, cell cycle progression, protein quality control, oncogenesis, apoptosis, and stress response. Dysfunction of the UPP is associated with many diseases, including cancer, neurodegeneration, autoimmune and inflammatory response, and infectious diseases [1,16,17,18,19]. As a result of its key functional role, the UPP represents an attractive drug target that has been extensively investigated during the last two decades [20,21,22,23,24,25,26]. Many structurally diverse inhibitors of proteasomes, both synthetic and natural products, have been discovered [27,28,29,30].

The presence of the ubiquitin–proteasome pathway has been demonstrated in animals [1], plants [31] and yeast [32]. In our laboratory, we identified the 26S proteasomes in lignin-degrading fungi *Trametes versicolor* [33] and *Phlebia radiata* [34], thus providing the first report on proteasomes in fungi other than yeast. Moreover, previous studies from our laboratory have shown that the ubiquitin-proteasome pathway is involved in the regulation of ligninolytic enzymes of white-rot fungi (such as laccase, benzenediol:oxygen oxidoreductase, EC 1.10.3.2) upon nutrient starvation [34,35,36] and in response to Cd^2+^ exposure [33] and ER stress [37]. Lignin-degrading Basidiomycetes are increasingly being investigated due to their ecological significance and various potential applications of ligninolytic enzymes [38,39], especially laccase [40,41], in several industrial and biotechnological processes, including the application in the pulp and paper industry [42], enzymatic bioremediation [43], biosensor [44] and biofuel cell [45] construction, and medicine [46,47]. Numerous studies indicated that laccase production can be considerably stimulated by the presence of inducers, mainly aromatic or phenolic compounds related to lignin or lignin derivatives, such as ferulic acid, 2,5-xylidine, p-anisidine, or veratryl alcohol [48,49,50,51]. In addition to these inducing substances, several heavy metal ions such as copper and cadmium have been found to stimulate laccase activity in white-rot fungi. We have previously shown that blocking of proteasome-mediated degradation with specific inhibitors leads to significant reduction of Cd^2+^-stimulated laccase activity in Cd^2+^-supplemented cultures of *T. versicolor* [33].

Ferulic acid, a polyphenol compound ubiquitous in plants, is known to induce laccase activity in liquid cultures of white-rot fungi when added at concentrations in a range of from 0.2 mM to 1 mM [50,52,53,54]. Polyphenols are classified into different groups based on the number of phenolic rings they contain as well as the structural moiety that holds these rings together [55]. The main groups include: flavonoids, phenolic acids (hydroxybenzoic acids, hydroxycinnamic acids), phenolic alcohols, and less common stilbenes and lignans [55,56]. Ferulic acid belongs to hydroxycinnamic acid derivatives of a group of phenolic acids [55]. In recent years, several lines of evidence have shown that ferulic acid acts as a potent antioxidant due to its ability to scavenge free radicals and enhance a cell stress response through the up-regulation of cytoprotective enzymes. Ferulic acid has also been shown to inhibit the expression and/or activity of cytotoxic enzymes [57,58,59,60].

Recent studies have shown that some natural polyphenols like flavonoids can modulate the functionality of the proteasome [61,62]. The goal of the current study was to determine the effect of ferulic acid (4-hydroxy-3-methoxycinnamic acid), a phenolic inducer of fungal laccase, on proteasomes isolated from mycelia of the wood-decomposing basidiomycete *T. versicolor*. Our results indicate that ferulic acid at concentrations known to induce laccase activity in the white rot fungus *T. versicolor* has an inhibitory effect on the 26S proteasomes in vitro. The present study provides new insights into the role of this phenolic acid in lignin-degrading fungi.

## 2. Results and Discussion

### 2.1. Comparison of the Effect of Various Phenolic Acids on CHTL Activity of T. versicolor Proteasomes

**Figure 1:**
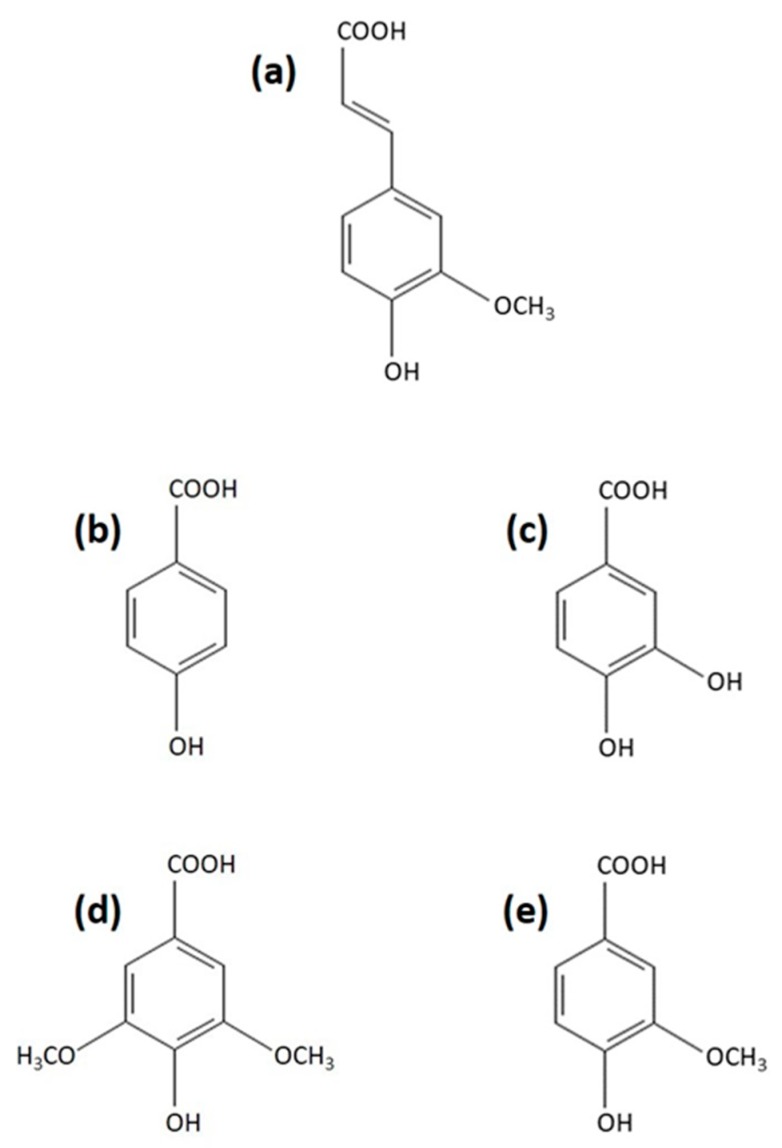
Chemical structure of: (**a**) ferulic acid, (**b**) p-hydroxybenzoic acid, (**c**) protocatechuic acid, (**d**) syringic acid, and (**e**) vanillic acid.

As an initial experiment, we assayed the effect of various phenolic acids (Figure 1), known to induce laccase activity in cultures of the white-rot basidiomycete *T. versicolor* [50,51], on the chymotrypsin-like activity of proteasomes isolated from mycelia of this fungus (Table 1). The 26S proteasomes were separated from low molecular proteases with a modified method proposed by Staszczak and Jarosz-Wilkołazka [33] adapted from [63], using a 500 kDa cut-off membrane (see Section 3.4). The proteasome preparations were preincubated with ferulic acid (4-hydroxy-3-methoxycinnamic acid), p-hydroxybenzoic acid, protocatechuic acid (3,4-dihydroxybenzoic acid), syringic acid (3,5-dimethoxy-4-hydroxybenzoic acid), and vanillic acid (4-hydroxy-3-methoxybenzoic acid). The most commonly measured „signature” proteasome activity is the CHTL activity, which is also most sensitive to proteasome inhibitors [9,64]. Since the assembly and activity of the 26S proteasome is ATP-dependent [15,65], proteasomal peptidase activity against Suc-LLVY-AMC, which is a fluorogenic substrate specific for CHTL activity, was assayed in the presence of 2 mM ATP. From the tested compounds, only ferulic acid affected chymotrypsin-like activity of *T. versicolor* proteasomes. The preincubation of the 26S fungal proteasomes with ferulic acid at concentrations of 200 µM and 1 mM for 30 min reduced CHTL activity by 15% and 32%, respectively. In contrast to ferulic acid (Figure 1a) belonging to the hydroxycinnamic acid group of phenolic acids, none of the hydroxybenzoic acids (Figure 1b–e) used in this study (p-hydroxybenzoic, protocatechuic, syringic, or vanillic acid) exhibited proteasome inhibitory properties, even at high concentrations. The potent proteasome inhibitors MG132 (100 µM) and lactacystin-β-lactone (25 µM) used as comparison with the tested compounds reduced the CHTL activity of the fungal proteasomes by about 47% and 80%, respectively.

### 2.2. Effect of Ferulic Acid on 26S Proteasome Peptidase Activities

Based on the above findings, we sought to investigate whether ferulic acid was able to inhibit all three proteasome peptidase activities. The 26S proteasomes obtained from the mycelial extracts of *T. versicolor*, using a five-step separation procedure with 500 kDa membranes (see Section 3.4), were preincubated with ferulic acid at various concentrations for 30 min. Then, different fluorogenic peptide substrates specific for each active site: Suc-LLVY-AMC, Z-GGR-AMC, and Z-LLE-AMC were used for measuring chymotrypsin-like, trypsin-like, and caspase-like activity, respectively. Proteasome inhibitors: clasto-lactacystin β-lactone (25 µM) and MG132 (100 µM) were used as comparison with ferulic acid. These inhibitors are known to act predominantly on the chymotrypsin-like activity [21,66]. Their affinity toward CHTL active sites is at least two orders of magnitude higher than in the case of TL and CL activities [21].

**Figure 2:**
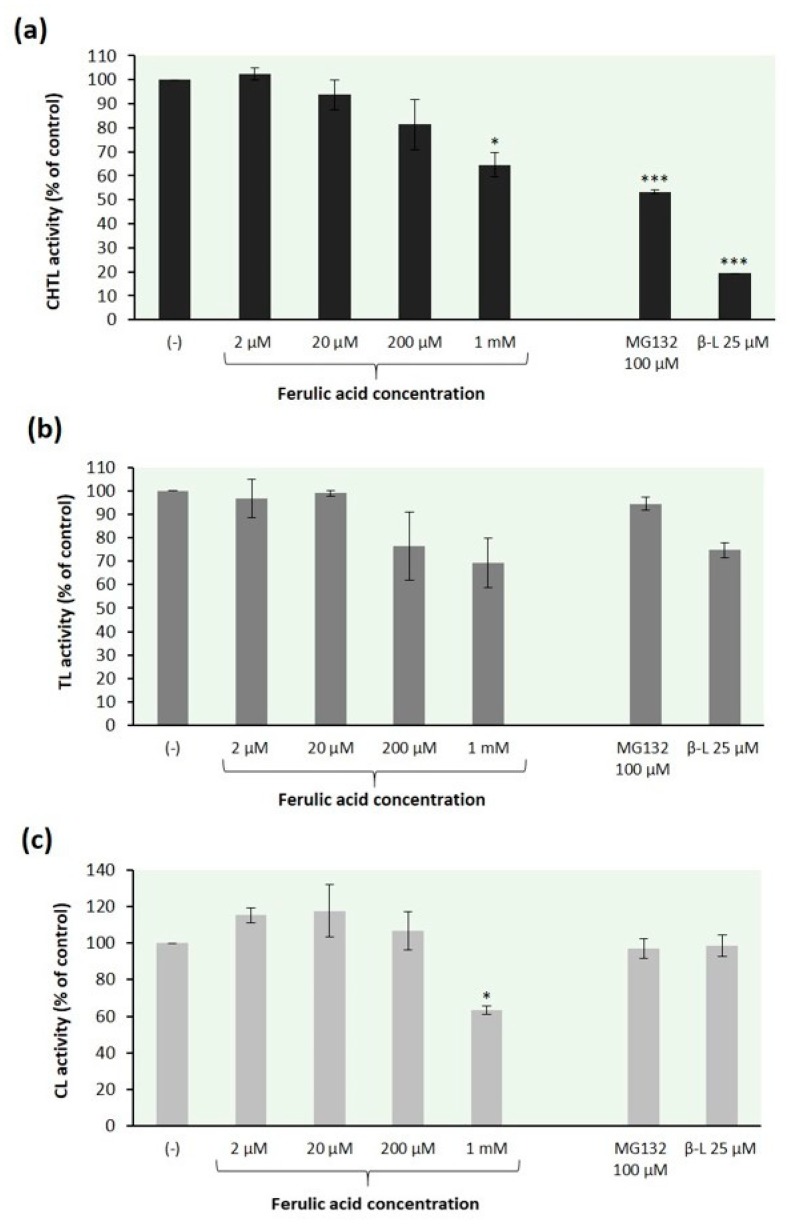
Effect of ferulic acid on peptidase activities of the 26S fungal proteasomes: (**a**) chymotrypsin-like (CHTL), (**b**) trypsin-like (TL), and (**c**) caspase-like (CL) activity. The 26S proteasomes were obtained from *T. versicolor* mycelial extracts using a 5-step separation procedure with 500 kDa cut-off membranes. Reaction mixtures containing the proteasome solution were preincubated with ferulic acid at various concentrations for 30 min before the addition of the fluorogenic peptide substrates: Suc-LLVY-AMC, Z-GGR-AMC and Z-LLE-AMC for measuring CHTL, TL, and CL activity, respectively. Proteasome inhibitors MG132 and clasto-lactacystin β-lactone (β-L) were used as comparison with ferulic acid. The specific activity (nanomoles of AMC per mg of protein) of samples with the DMSO solvent alone was taken to be 100%. The data represent means ±SD of three independent experiments performed in duplicate (* *p* < 0.05, ** *p* < 0.01, and *** *p* < 0.001).

The data in Figure 2 demonstrate that ferulic acid affected all peptidase activities of the 26S fungal proteasomes in a concentration-dependent manner. Our findings are consistent with a previous report that curcumin (diferuloylmethane), which is structurally related to ferulic acid, possesses the ability to inhibit all three peptidase activities of the purified proteasome to almost similar extent [67]. Ferulic acid inhibited the CHTL proteasome activity by 19% and 35% at concentrations of 200 µM and 1 mM, respectively (Figure 2a). Similar results were found for the relation between the TL activity of fungal proteasomes and the ferulic acid concentration; this proteasome peptidase activity was reduced by 24% and 31%, respectively (Figure 2b). In contrast to the CHTL and TL activity, the CL activity of *T. versicolor* proteasomes was slightly stimulated at low concentrations of ferulic acid but inhibited at concentration above 200 µM (Figure 2c). The preincubation of fungal proteasomes with this compound at concentration of 1 mM resulted in the inhibition of the CL activity by 37%. Such biphasic dose response has been previously reported for the effect of curcumin on chymotrypsin-like proteasomal activity of human keratinocytes [68].

**Figure 3:**
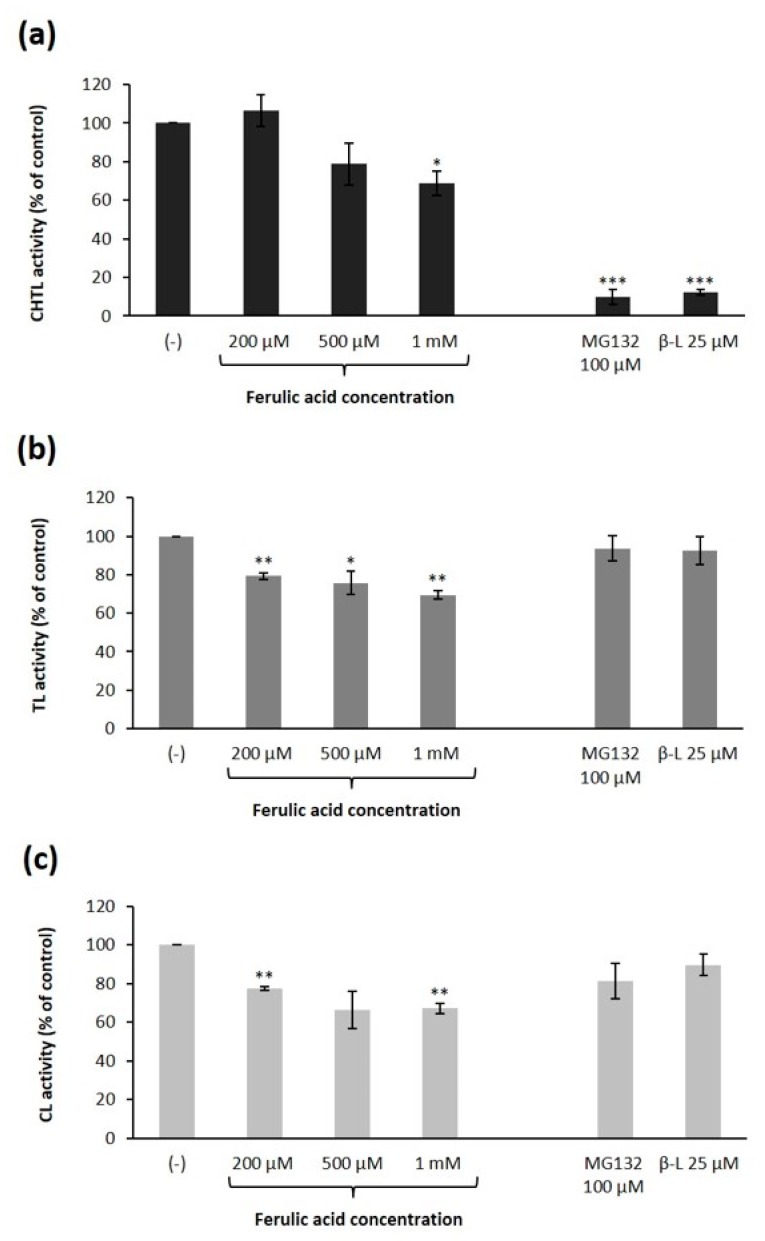
Effect of ferulic acid on peptidase activities of the 26S human proteasomes: (**a**) chymotrypsin-like (CHTL), (**b**) trypsin-like (TL), and (**c**) caspase-like (CL) activity. Reaction mixtures containing the 26S human proteasome solution were preincubated with ferulic acid at the indicated concentrations for 30 min before the addition of the fluorogenic peptide substrates: Suc-LLVY-AMC, Z-GGR-AMC and Z-LLE-AMC for measuring CHTL, TL, and CL activity, respectively. Proteasome inhibitors clasto-lactacystin β-lactone (β-L) and MG132 were used as comparison to ferulic acid. The specific activity (nanomoles of AMC per mg of protein) of samples with the DMSO solvent alone was taken to be 100%. The data represent means ±SD of three independent experiments performed in duplicate (* *p* < 0.05, ** *p* < 0.01, and *** *p* < 0.001).

For comparison purposes, the evaluation of the inhibitory activity of ferulic acid (at concentrations no less than 200 µM) was also performed with the human 26S proteasomes using specific substrates for the CHTL, TL, and CL activity of the proteasome (Figure 3). These experiments revealed that ferulic acid inhibited the peptidase activities of the human proteasome in a dose-dependent manner. The CHTL activity was reduced by this phenolic acid at concentrations above 200 µM. Ferulic acid inhibited the CHTL proteasomal activity by 21% and 31% at concentrations of 500 µM and 1mM, respectively (Figure 3a). The preincubation of the human 26S proteasomes with ferulic acid at concentrations of 200 µM, 500 µM, and 1 mM resulted in the inhibition of TL activity by 21%, 24%, and 30%, respectively (Figure 3b). A similar extent of inhibition was observed for CL proteasome activity (Figure 3c). Human proteasomes have been found to be slightly more sensitive to ferulic acid compared to those of fungi.

### 2.3. Degradation of a Full-Length Protein Substrate by 26S Fungal Proteasomes

In an independent experiment we examined the ability of ferulic acid to inhibit the 26S proteasome-catalyzed breakdown of a full-length protein. Relatively simple methods have been developed to measure the absolute rates of protein degradation by 26S proteasome complexes without the use of isotopes or ubiquitination [15,69]. It has been shown that ATP binding alone supports 19S–20S association, gate opening for substrate entry, and translocation of unfolded substrates into the proteasome [15,70]. When incubated with 26S proteasomes, proteins that lack distinct tertiary structures (e.g., casein or oxidized ovalbumin) are hydrolyzed without ubiquitination by the 26S proteasomes in an ATP-dependent manner for many hours [15,69]. Peptide bond cleavage in proteins is then assayed by measuring the appearance of new amino groups with fluorescamine. This approach is a good alternative to procedures based on the use of radioisotope-labeled protein substrates.

**Figure 4:**
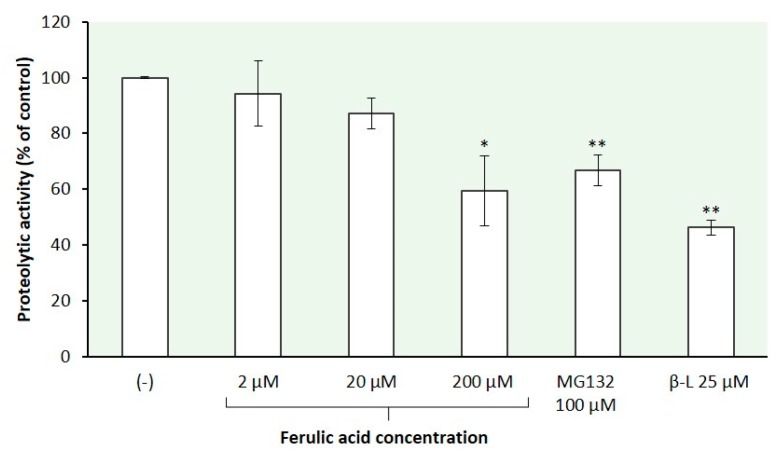
Effect of ferulic acid on the absolute rate of protein degradation by the 26S proteasomes isolated from *T. versicolor*. Reaction mixtures containing the proteasome solution were preincubated with ferulic acid at various concentrations for 30 min before the addition of a model protein substrate (β-casein). Proteasome inhibitors MG132 and clasto-lactacystin β-lactone (β-L) were used for comparison. The rate of protein degradation was spectrofluorometrically quantified by the fluorescamine assay for release of free α-amino groups generated upon the cleavage of peptide bonds in β-casein. The control reaction mixture contained the DMSO solvent. The data represent means ±SD of four independent experiments performed in duplicate (* *p* < 0.05 and ** *p* < 0.01).

Here, we used β-casein as the model unfolded substrate in the study of protein degradation catalyzed by the 26S proteasomes isolated from mycelia of *T. versicolor*. The proteasome preparations were preincubated for 30 min with ferulic acid at concentrations of 2 µM, 20 µM, and 200 µM before the addition of the substrate protein. Proteasome inhibitors MG132 (100 µM) and clasto-lactacystin β-lactone (25 µM) were used as comparison to ferulic acid. Casein in its native form has little tertiary structure and can be degraded in an ATP-dependent linear manner in the absence of ubiquitination [9,69]. The rate of protein degradation was spectrofluorometrically quantified by the modified fluorescamine assay [9] as described in Section 3.6.1. Figure 4 summarizes the results of this experiment. Ferulic acid blocked proteolysis in a concentration-dependent manner. The preincubation of the fungal proteasomes with this phenolic acid at concentrations of 2 µM, 20 µM, and 200 µM reduced the rate of β-casein degradation by 6%, 13%, and 41%, respectively. The latter value is higher than that determined in the present study for the effect of 200 µM ferulic acid on each of the proteasome peptidase activities (CHTL, TL, and CL activity) and reflects the contribution of the different active sites in protein breakdown [9]. Our findings are consistent with previous reports that inactivation of one type of proteasomal active site is not sufficient to markedly block protein degradation, that the CHTL site is not rate-limiting for many proteins, and that significant inhibition of protein breakdown is observed only when the CHTL sites and either the TL or CL sites are also inhibited [9]. Thus, the TL and CL sites play a significant, generally unappreciated role in protein degradation.

**Figure 5:**
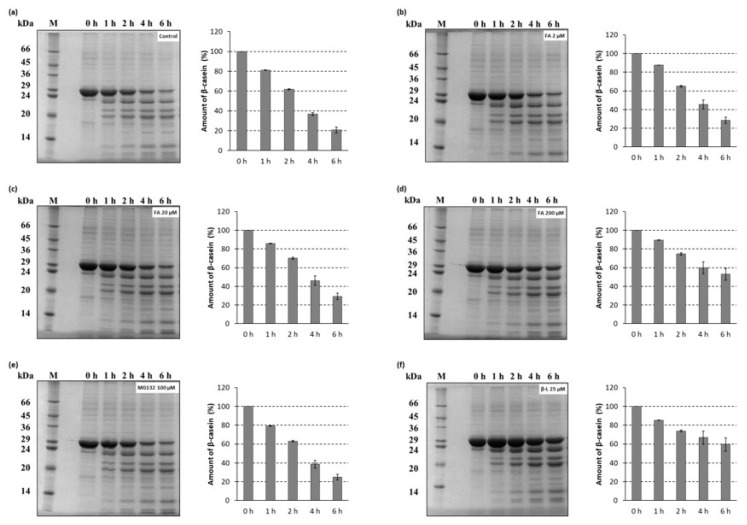
Time course of the 26S fungal proteasome catalyzed β-casein degradation. Proteolysis reaction mixtures containing the 26S proteasomes isolated from *T. versicolor* were preincubated with: (**a**) DMSO as solvent control or (**b**–**d**) ferulic acid (FA) at various concentrations for 30 min before the addition of a model protein substrate (β-casein). Proteasome inhibitors: (**e**) MG132 and (**f**) clasto-lactacystin β-lactone (β-L) were used as comparison to ferulic acid. At the indicated time points reaction aliquots were added to SDS sample buffer and analyzed by SDS-PAGE (12% polyacrylamide gel, 29:1 acrylamide:bisacrylamide) followed by microwave-assisted Coomassie Brilliant Blue R-250 staining. The data represent results from one of three independent experiments with similar results. Quantification plots of the PAGE gels illustrate the amount of β-casein.

Degradation of a full-length protein (β-casein) by the 26S proteasomes isolated from *T. versicolor* was also determined by a gel-electrophoretic analysis (Figure 5). Aliquots of the proteolysis mixtures were taken at different times within 6 h and resolved on 12% SDS-PAGE as described in Section 3.6.2. Expectedly, in an additional control reaction, no protein degradation was observed when the proteasomes were omitted from the reaction (Figure S1). The ability of ferulic acid to inhibit the rate of degradation of β-casein in a dose-dependent manner was confirmed by the gel-electrophoretic analysis. It is worth noting that ferulic acid at the concentration known to induce laccase activity in the white-rot fungi inhibited the proteasome-mediated proteolysis more strongly (Figure 5d) than MG132 used as comparison (Figure 5e). This is consistent with the results of the fluorescamine assay.

## 3. Materials and Methods

### 3.1. Materials

Ferulic acid (4-hydroxy-3-methoxycinnamic acid), p-hydroxybenzoic acid, vanillic acid (4-hydroxy-3-methoxybenzoic acid), β-casein from bovine milk, Suc-Leu-Leu-Val-Tyr-7-amido-4-methylcoumarin (Suc-LLVY-AMC), Z-Gly-Gly-Arg-7-amido-4-methylcoumarin (Z-GGR-AMC), Z-Leu-Leu-Glu-amido-4-methylcoumarin (Z-LLE-AMC), fluorescamine, Z-Leu-Leu-Leu-al (MG132), clasto-lactacystin β-lactone, dimethyl sulfoxide (DMSO), MgCl_2_, ATP, glycerol, 7-amino-4-methylcoumarin (AMC), L-leucine, Bradford Reagent, acrylamide/bis-acrylamide 30% (29:1), sodium dodecyl sulfate (SDS), and Dalton Mark VII-L Standard Mixture 14,000–66,000 for SDS-PAGE were obtained from Sigma (St. Louis, MO, USA). Protocatechuic acid (3,4-dihydroxybenzoic acid) and syringic acid (3,5-dimethoxy-4-hydroxybenzoic acid) were obtained from Fluka (Buchs, Switzerland). The Human 26S Proteasome was purchased from BostonBiochem (Cambridge, MA, USA). All aqueous solutions were prepared using water purified with Milli-Q system (Millipore, Milford, CT, USA). All other chemicals were of analytical grade. Biomax PBVK polyethersulfone ultrafiltration membranes (NMWL 500 kDa) and Amicon nitrogen-pressure based concentration apparatus were obtained from Millipore (Bedford, MA, USA).

### 3.2. Organism and Culture Conditions

*Trametes versicolor* (ATCC44308, strain FCL 7) was grown as described previously [35,71]. Briefly, mycelia were maintained, as surface cultures at 26 °C, in scintillation vials containing 10 mL of growth medium prepared according to Fåhraeus and Reinhammar [72]. After the 7-day cultivation period, fungal mycelia were harvested and frozen (at −20 °C) until use.

### 3.3. Preparation of Crude Mycelium Extract

Crude mycelial extracts were prepared essentially as described previously [36]. Briefly, mycelia were allowed to defrost on ice and then homogenized in an ice-chilled motor-driven Potter’s homogenizer (Universal Laboratory Aid type 309, Mechanika Precyzyjna, Warsaw, Poland), in 0.75 mL (per one mycelium) of 50 mM Tris-HCl buffer, pH 7.3, containing 5 mM MgCl_2_. The homogenates were then centrifuged at 10,000× *g* for 10 min at 4 °C (Sigma 3K18, Sigma Laborzentrifugen GmbH, Osterode am Harz, Germany). The supernatant fractions were aliquoted into single-use aliquots, frozen and stored at −20 °C until use for separation of proteasomes.

### 3.4. Isolation of 26S Proteasomes

The method used to separate proteasomes from other low molecular proteases, adapted from [63], followed that described previously with slight modifications [33]. Briefly, frozen aliquots (0.5 mL) of crude mycelial extracts were thawed on ice and brought to 20 × their volumes in separation buffer (50 mM Tris–HCl, pH 8.0, 2 mM ATP, 5 mM MgCl_2_, and 20% glycerol). The diluted samples were then concentrated to the original volumes using Biomax PBVK polyethersulfone ultrafiltration membranes (NMWL 500 kDa) and Amicon nitrogen-pressure based concentration apparatus (Millipore, Bedford, MA, USA). This step was repeated 5 times (if not stated otherwise) using freshly prepared separation buffer each time. All steps were carried out on ice. Retentates containing high molecular weight fractions (above 500 kDa) were aliquoted into single-use aliquots and stored at −20 °C until being assayed for proteasome activities.

### 3.5. Assays of 26S Proteasome Peptidase Activities

Peptidase activities of the isolated fungal 26S proteasomes were assayed by measuring the hydrolysis of fluorogenic peptide substrates specific for each active site: Suc-LLVY-AMC, Z-GGR-AMC and Z-LLE-AMC for chymotrypsin-like, trypsin-like, and caspase-like activity, respectively. The modified SDS-stopped method [73,74] was performed essentially as described previously with slight modifications [34]. Briefly, assay mixtures containing the 26S proteasomes at a concentration of 40 µg protein/mL, 100 μM peptide substrate (in DMSO), appropriate dilution of the stock ferulic acid solution (to yield a final concentration of: 2 µM, 20 µM, 200 µM, 1 mM), 100 mM Tris–HCl buffer (pH 8.0), 2 mM ATP, and 5 mM MgCl_2_, were made up in a total volume of 100 μL and then incubated at 37 °C for 2 h. The reactions were stopped by addition of 100 μL of 10% SDS (*w*/*v*) and 2 mL of 100 mM Tris–HCl buffer, pH 9.0. Control reaction mixtures contained an equivalent amount of DMSO (solvent for the tested compounds). A blank lacking the proteasomes was run in parallel. Proteasome inhibitors clasto-lactacystin β-lactone (25 µM) and MG132 (100 µM) were used as comparison to ferulic acid. The fluorescence of liberated 7-amido-4-methylcoumarin (AMC) was quantified in a spectrofluorometer (FluoroMax-2, Instruments S.A., Inc., JOBINYVON/SPEX Division with FluorEssence software version 3.0, Horiba Scientific, Edison, NJ, USA), with an excitation wavelength of 360 nm and an emission wavelength of 440 nm. The excitation and emission slits were 1 and 0.2, respectively. The amount of released AMC was calculated using a standard curve prepared with known dilutions of AMC in 5% DMSO in 100 mM Tris–HCl buffer, pH 8.0. Measurements at 360 nm excitation/440 nm emission were included for each ferulic acid concentration tested to subtract the intrinsic fluorescence of ferulic acid [75]. The standard curve was determined for each experiment. Specific peptidase activity was expressed in nanomoles of AMC released per milligram of protein per 2 h. Assays with the human 26S proteasome (0.185 nM) were performed for comparison. Other experimental details are provided in figure legends and table footnotes.

### 3.6. Degradation of a Full-Length Protein Substrate by 26S Proteasomes

Degradation of a full-length protein (β-casein) was determined by a fluorescamine assay and by a gel-electrophoretic analysis.

#### 3.6.1. Fluorescamine Assay

The rate of protein degradation was spectrofluorometrically quantified with the modified fluorescamine assay [9] for release of free α-amino groups generated upon the cleavage of peptide bonds in β-casein, which was used as a model unfolded protein substrate. Proteolysis of β-casein was performed by incubation of the protein substrate (0.5 mg/mL) with the isolated fungal 26S proteasomes (36 µg/mL) at 37 °C in 100 mM Tris–HCl buffer (pH 8.0) containing 2 mM ATP and 5 mM MgCl_2_, in the absence or presence of ferulic acid (in a final concentration up to 200 µM). The control reaction mixture contained an equivalent amount of DMSO (solvent for the tested compounds). A blank lacking the proteasomes was run in parallel. Proteasome inhibitors clasto-lactacystin β-lactone (25 µM) and MG132 (100 µM) were used as comparison to ferulic acid. Reaction aliquots were taken at 0, 2 and 4 h, and proteolysis was stopped by the addition of HClO_4_ (to a final concentration of 6%). The mixtures were vortexed (REAX top, Heidolph Instruments, Schwabach, Germany) thoroughly and kept on ice for 30 min prior to centrifugation for 15 min at 20,000× *g*, at 4 °C (Sigma 3K18, Sigma Laborzentrifugen GmbH, Osterode am Harz, Germany). Then 40 µL samples (in duplicates) were mixed with 1.86 mL of 0.1 M sodium phosphate buffer (pH 6.8) and 100 µL of a freshly prepared fluorescamine solution (0.03% in acetone). The mixture was then vortexed for 1 min. Fluorescence of adducts formed by fluorescamine with N-termini of peptides, generated by proteasomal cleavage was measured using an excitation wavelength of 370 nm and an emission of 475 nm (FluoroMax-2, Instruments S.A., Inc., JOBINYVON/SPEX Division with FluorEssence software version 3.0, Horiba Scientific, Edison, NJ, USA). A standard curve for leucine was used to calibrate the assay.

#### 3.6.2. SDS-PAGE

Degradation assays were carried out with β-casein as a model unfolded protein substrate. Proteolysis reaction mixtures containing, in a total volume of 150 µL, 100 mM Tris–HCl buffer (pH 8.0), 2 mM ATP, 5 mM MgCl_2_, isolated fungal 26S proteasomes at a concentration of 58 µg/mL, β-casein (0.5 mg/mL), and appropriate dilution of the stock ferulic acid solution (to yield final concentration of: 2 µM, 20 µM, 200 µM) were incubated at 37 °C. The control reaction mixture contained an equivalent amount of DMSO (solvent for tested compounds). Reactions with proteasome inhibitors clasto-lactacystin β-lactone (25 µM) and MG132 (100 µM) were run in parallel for comparison. At 0, 1, 2, 4 and 6 h mixture aliquots of 20 µL were added to 5 × SDS sample buffer and analyzed by SDS-PAGE (Minigel-Twin, Biometra GmbH, Gӧttingen, Germany) using a 4% stacking gel and a 12% separating gel (29:1 acrylamide:bisacrylamide) in the Laemmli’s discontinuous buffer system [76]. The samples were run at 150 V for ~1.5 h at room temperature. The rate of protein degradation was visualized by microwave-assisted Coomassie Brilliant Blue R-250 (Merck, Darmstadt, Germany) staining according to the modified method originally described by Wong et al. [77] and analyzed using a Syngene G:BOX HR gel documentation system (Syngene, Cambridge, UK) and GeneSnap software (version 6.08.04, Synoptics Ltd; Cambridge, UK).

### 3.7. Protein Determination

Protein concentrations were determined with the Bradford method [78] using bovine serum albumin as a standard.

### 3.8. Statistical Analysis

Where appropriate, data were analyzed using the Student’s *t* test (* *p* < 0.05, ** *p* < 0.01, and *** *p* < 0.001). Data represent means ±SD of at least three independent experiments performed in duplicate.

## 4. Conclusions

Our results indicate that ferulic acid at concentrations known to induce laccase activity in the white-rot fungus *T. versicolor* has inhibitory effect on the 26S proteasomes in vitro. The present study provides new insights into the role of this phenolic acid in lignin-degrading fungi. However, the detailed molecular mechanisms involved remain to be elucidated by future research.

## Figures and Tables

**Table 1:** In vitro effect of various phenolic acids on chymotrypsin-like activity of *T. versicolor* proteasomes.

Compound	Final Concentration (mM)	Remaining CHTL Activity ^1^ (% of Control)
None	-	100
Ferulic acid	0.002	101
	0.02	99
	0.2	85
	1	68
p-Hydroxybenzoic acid	0.002	101
	0.02	98
	0.2	94
	1	100
Protocatechuic acid	0.002	105
	0.02	98
	0.2	92
	1	103
Syringic acid	0.002	102
	0.02	104
	0.2	105
	1	102
Vanillic acid	0.002	107
	0.02	107
	0.2	96
	1	100

^1^ The 26S proteasomes were obtained from *T. versicolor* mycelial extracts using a 1-step separation procedure with 500 kDa cut-off membranes. Aliquots of the 26S fungal proteasome solution were preincubated with phenolic acids at the indicated concentrations for 30 min before the addition of the fluorogenic peptide substrate (Suc-LLVY-AMC) for measuring chymotrypsin-like (CHTL) activity. The specific activity (nanomoles of AMC per mg of protein) of samples with the DMSO solvent alone was taken to be 100%. The data represent means of at least three independent experiments with SD not exceeding 10% of the mean.

## Supplementary Materials

Supplementary materials can be found at https://www.mdpi.com/1422-0067/21/7/2463/s1. Figure S1: Control reaction of β-casein degradation with no proteasome added.

## Abbreviations

AMC	7-amino-4-methylcoumarin
CHTL	chymotrypsin-like
CL	caspase-like
DMSO	dimethyl sulfoxide
MG132	Z-Leu-Leu-Leu-al
Suc-LLVY-AMC	Suc-Leu-Leu-Val-Tyr-7-amido-4-methylcoumarin
TL	trypsin-like
UPP	ubiquitin-proteasome pathway
Z-GGR-AMC	Z-Gly-Gly-Arg-7-amido-4-methylcoumarin
Z-LLE-AMC	Z-Leu-Leu-Glu-amido-4-methylcoumarin
